# Machine learning algorithms to predict depression in older adults in China: a cross-sectional study

**DOI:** 10.3389/fpubh.2024.1462387

**Published:** 2025-01-07

**Authors:** Yan Li Qing Song, Lin Chen, Haoqiang Liu, Yue Liu

**Affiliations:** ^1^College of Sports, Nanjing Tech University, Nanjing, China; ^2^School of Athletic Performance, Shanghai University of Sport, Shanghai, China

**Keywords:** depression, machine learning, health promotion, CHARLS, China

## Abstract

**Objective:**

The 2-fold objective of this research is to investigate machine learning's (ML) predictive value for the incidence of depression among China's older adult population and to determine the noteworthy aspects resulting in depression.

**Methods:**

This research selected 7,880 older adult people by utilizing data from the 2020 China Health and Retirement Longitudinal Study. Thereafter, the dataset was classified into training and testing sets at a 6:4 ratio. Six ML algorithms, namely, logistic regression, k-nearest neighbors, support vector machine, decision tree, LightGBM, and random forest, were used in constructing a predictive model for depression among the older adult. To compare the differences in the ROC curves of the different models, the Delong test was conducted. Meanwhile, to evaluate the models' performance, this research performed decision curve analysis (DCA). Thereafter, the Shapely Additive exPlanations values were utilized for model interpretation on the bases of the prediction results' substantial contributions.

**Results:**

The range of the area under the curve (AUC) of each model's ROC curves was 0.648–0.738, with significant differences (*P* < 0.01). The DCA results indicate that within various probability thresholds, LightGBM's net benefit was the highest. Self-rated health, nighttime sleep, gender, age, and cognitive function are the five most important characteristics of all models in terms of predicting the occurrence of depression.

**Conclusion:**

The occurrence of depression among China's older adult population and the critical factors leading to depression can be predicted and identified, respectively, by ML algorithms.

## Introduction

The Diagnostic and Statistical Manual of Mental Disorders, Fifth Edition (DSM-5) defines depression as a persistent state of low mood accompanied by various psychological and physiological symptoms, significantly impairing an individual's daily functioning. Among the older adult, depression is a typical and serious issue concerning mental health (1). Given that about 300 million people globally are affected by depression, this condition is among the general population's most common mental illnesses and in the top three of the causes of disability (2, 3). In terms of global disease burden, the projection is that depression will be ranked first by 2023 (3). According to accumulating evidence, depression among the older adult is suggested to be a potential risk factor for the start of diseases related to aging, including dementia, cardiovascular, and metabolic diseases (4, 5). Depression that is mild and severe are substantially related with considerably high rates of suicide (6, 7), low-quality life (8), high death rates (9), and an increase in the cost of health care (8). In addition, inadequacy in treating depression among the older adult has been observed (10, 11). Although depression is viewed to be typically related to aging, concerns have been raised whether or not treatments for depression are effective (12). In 2021, a mere 0.5% of patients in China suffering from depression received sufficient treatment, according to the China Mental Health Survey (13). To identify high-risk people and implement appropriate early interventions, a crucial undertaking is risk prediction for depression (14). Therefore, preventing and treating depression among the older adult necessitate the identification of the characteristics and risk factors of this condition.

Machine learning (ML) has achieved critical developments in recent years, particularly in mental health diagnosis (15). ML is a crucial computer science branch, the objective of which is the performance improvement of a variety of tasks (e.g., prediction) via pattern extraction from data. To comprehend the mental disorder properties and the related risk and prognostic factors, researchers in clinical psychology and psychiatry are extensively performing wide-ranging and varied evaluations, including self-report measures, physiological factors, and imaging data. Current datasets normally comprise thousands of measurements often gathered frequently over time. ML methods can be utilized for complex data structures, thereby enabling an improved understanding and conceptualization of mental disorders, detection and prediction of symptoms' risk and trajectory, and investigation of outcomes of treatments and differential treatment responses. A research conducted data gathering from 1,617 participants in the China Health and Retirement Longitudinal Study (CHARLS) registry from 2011 to 2018, using three data balancing techniques and four ML algorithms to build predictive models for classifying depression prognosis (16). In another research, depression-related data were used from 2,548 home-dwelling Chinese older adult individuals in CHARLS (2011–2018); depression outcomes were estimated by utilizing three ML classification algorithms: gradient boosting decision tree (DT), support vector machine (SVM), and random forest (RF) (17). Even though prior research has developed models on depression risk prediction by utilizing longitudinal questionnaire data, information obtained in previous studies were gathered pre-COVID-19 pandemic (18). Evidently, society had been severely impacted by the pandemic, including the people's lifestyle and health status. According to studies, a correlation exists between the COVID-19 pandemic and an increase in depression symptoms (19, 20). A research conducted regarding the occurrence of depression in the older adult in low- and middle-income countries indicates that the incidence of symptoms of depression reported at the height of the COVID-19 pandemic was comparatively higher than pre-pandemic estimates (21, 22). In a research assessing changes in the symptoms of depression among older adult type 2 diabetes patients in the US from 2016 to 2020, the results indicated that the pervasiveness of mild or markedly severe symptoms of depression among these patients during the COVID-19 pandemic was over 1.6 times higher than prior to the pandemic (23).

The current research analyzed the updated CHARLS data gathered during the COVID-19 pandemic. The reason for selecting this dataset is that it substantially embodies depression's characteristics at this stage among the older adult. To predict depression and determine the related influencing factors leading to this condition among the older adult in China, the following six models were established: k-nearest neighbors (KNN), logistic regression, DT, RF, LightGBM, and SVM.

## Research data and methods

### Data sources

CHARLS was the first nationally representative survey of middle-aged and older adult people in China and used the multi-stage, stratified probability proportional to size (PPS) sampling method to select samples. The PPS sampling method guarantees the reasonable representation in the sample of individuals from different regions and of varying types (24). Through face-to-face interviews, including the use of questionnaire surveys and biomarker collection, data were collected through CHARLS. Investigators with professional training often conduct surveys, presenting data from a variety of modules, including sociodemography, lifestyle habits, psychological status, and health conditions, thereby guaranteeing data accuracy and consistency. This method presents dependable data support for developing a model for depression prediction for China's older adult. The Biomedical Ethics Committee of Peking University approved the 2020 CHARLS survey (approval number IRB00001052-11015.1.2). For the field survey, respondents who agreed to participate in the survey were asked to sign two informed consent forms; one copy for the respondents and the other is kept in the CHARLS office. Before being released, all data were strictly cleaned and anonymized. A follow-up survey every 2 years is planned by the CHARLS survey, the objective of which was to gather data from middle-aged and older adult people in China over time. However, the 2020 CHARLS data are the latest publicly released data owing to the time required for data collection, cleaning, verification, and release. Accordingly, the current study uses the 2020 CHARLS data. Criteria for inclusion are as follows: (1) individuals aged ≥ 60 years and (2) data on whether or not there are explanatory variables for the depression dimension. Criteria for exclusion are as follows: (1) people aged < 60 years and (2) over 10% missing values in the variables of interest. Moreover, replacing missing values (< 10%) entails using the median method.

### Outcome variable

To screen depressive symptoms, CHARLS uses as a common tool the Center for Epidemiological Studies Depression Scale-10 (CESD-10) from the Chinese Center for Epidemiological Studies (25). CESD-10 is selected to test the respondents' depressive symptoms. Moreover, CESD-10, which is reliable and valid, is appropriate for use in large-scale epidemiological surveys. A total of 10 questions are included in the questionnaire to test the past week's individual depressive symptom frequency. The questions include various facets, including depressive mood, sleep disorders, appetite changes, loss of interest, fatigue, difficulty concentrating, and feelings of inferiority (26). A four-level scoring system is typically used for the answers: rarely (under 1 day), sometimes (1–2 days), more than half the time (3–4 days), and almost every day (5–7 days). On the bases of the respondents' answers, each question's scores will be added to obtain the total CESD-10 score, in which the range of the total score is 0–30 points. Total scores of ≥10 or < 10 points indicate having and no depressive symptoms, respectively. The higher the score, the more severe the depressive symptoms (27).

### Sociodemographic factors

Variables for the sociodemographic data selected by this study comprise age, gender, education level, region, marital status, and living alone. Moreover, the variables were categorized as follows: gender: female or male; education level: illiterate, primary school, or junior high school and above; region: urban or rural; marital status: married, divorced, widowed, or unmarried; and living alone: yes or no. Age is considered a continuous variable.

### Health status

This study chose the following variables for health status: self-rated health (28), multimorbidity (29), Katz-ADL score (30), and Lawton-IADL score (30). The variables were specifically categorized as follows: self-rated health: very poor, poor, fair, good, or very good; and multimorbidity: yes or no. Meanwhile, Katz-ADL and Lawton-IADL scores were used to assess independent living ability and instrumental daily living function, respectively, in which higher scores indicate poorer ability. The Katz-ADL and Lawton-IADL scores are considered continuous variables.

### Behavioral factors

Smoking, drinking, nighttime sleep, nap time, social activities, and weekly physical exercise are included in the behavioral factors. Nighttime sleep is categorized as short (< 6 h), moderate (6–8 h; reference group), and long (>8 h) (31, 32). Nap time is evaluated based on the following question: “How long do you usually nap in the past month?” The respondents were divided into 4 groups: no nap (0 min), short nap (< 30 min), moderate nap (30–90 min), and long nap (>90 min) (33). Smoking, drinking, and social activities are categorized as yes or no. Lastly, weekly physical exercise is categorized as 0 times per week, 1–4 times per week, or more than 4 times per week.

### Mental health factors

Cognitive function scores are included in the mental health factors. The American Health and Retirement Study (HRS) methods are used to measure the cognitive function scores (34). The participants underwent face-to-face assessments from four dimensions of cognitive function: orientation, memory, calculation, and drawing. The total cognitive score is the sum of orientation (five points), calculation (five points), memory (20 points), and drawing (one point), with a total score of 31 points (35).

### Statistical analysis

SPSS23.0 and Python3.8 were used for data analysis. Continuous variables were characterized. In addition, the Kolmogorov-Smirnov test was performed to determine if the normal distribution was followed by the test data. Meanwhile (x ± s), depicted the econometrics conforming to the normal distribution. *T*-test and analysis of variance (ANOVA) were used for mean comparisons between two groups and across multiple groups, respectively. M(Q1, Q3) indicates measures not adhering to a normal distribution or variance. For comparisons within or between groups, rank-sum tests were utilized. In addition, *n* (%) depicted categorical data. Lastly, chi-square or exact Fisher's test was utilized for analyzing across at least two groups, with a statistically significant difference of *P* < 0.05.

**Figure 1:**
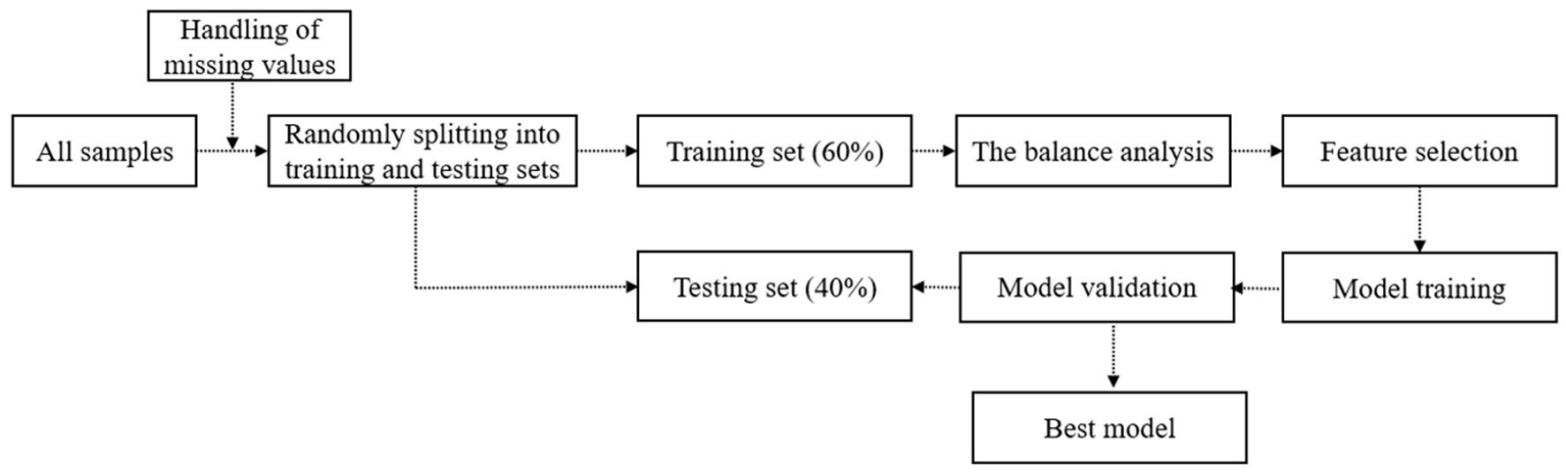
Schematic diagram of the construction of depression prediction framework.

The schematic of the depression prediction framework is presented in Figure 1, which outlines the different steps comprising data preprocessing and model development. Data were divided randomly into training and tests at a 6:4 ratio, thereby guaranteeing similar distribution across both datasets (36). Various methods were also used to fill in missing data values. This study specifically used the training set data's preprocessed data feature screening, in which imbalance was first addressed, followed by balance analysis. The data were then sampled t in the modeling for the unbalanced data. Thereafter, we utilized the SMOTE algorithm to oversample the training set's minority samples. The RF algorithm was utilized for feature selection as the classification model for recursive feature elimination. For the discrete variants, the chi-square detection was performed. For the continuous variables, point-two sequence correlation analysis was conducted. Eventually, characteristic variables with *P*-values below 0.05 were selected as model inputs.

Six models were constructed and forecast by this study: SVM, KNN, logistic regression, DT, RF, and LightGBM (Table 1). The ROC and AUC values via grid search and 5-fold cross-validation were utilized to evaluate the scores, resulting in the selection of the hyperparameters of the model. We selected evaluation metrics (e.g., precision, AUC area, sensitivity, true positives, and false positives) for the model's assessment on the training set. Thereafter, the Delong test was utilized for the comparison of the differences of the ROC curves across models. Decision curve analysis (DCA) was used to assess the performance of the model, in which the true and false positives of a model are used to compute an analytical measure called net benefit, thereby placing the advantages and disadvantages of the model on a comparable scale (37). This study used the Brier score, defined as the mean squared difference between the observed and predicted outcomes, to assess the top-performing model's probabilistic accuracy. In general, the range of the Brier score is from 0 to 1.00, with 0 indicating the best calibration (38). Given the ML model's black-box nature, the current research utilized the SHapely Additive exPlanations (SHAP) value to underscore the significance of the prediction results of the model. The summary graph provides a visual depiction of the contribution of each feature to the prediction results of the model.

**Table 1:** Comparison of different machine learning models.

**Model**	**Advantages**	**Disadvantages**	**Reasons for use/not use**
Support Vector Machine (SVM)	Suitable for small sample, high-dimensional data, high accuracy	Computationally intensive, especially with large datasets	Used in this study for its robustness and effectiveness in classification
k-Nearest Neighbors (KNN)	Simple and easy to implement, no assumptions about data distribution	Performance degrades with large datasets and high dimensions	Used in this study for its simplicity and effectiveness in small datasets
Logistic Regression	Simple model, easy to interpret, suitable for binary classification	Assumes a linear relationship between features and log odds	Used in this study for its interpretability and ease of implementation
Decision Tree (DT)	Easy to interpret, handles non-linear relationships	Prone to overfitting	Used in this study for its simplicity and interpretability
Random Forest (RF)	Reduces overfitting by averaging multiple decision trees, improves accuracy	Requires more computational resources	Used in this study for its balanced performance and feature selection
LightGBM	Efficient with large datasets, fast training	Sensitive to noisy data	Chosen for this study for its efficiency and speed
Artificial Neural Networks (ANN)	Captures complex patterns, highly flexible	Black-box nature, hard to interpret	Not used due to interpretability issues

## Findings

### Descriptive analysis results

This study's final effective sample includes 7,880 older adult people: 4,125 females (52.35%), with average age of 69.05 ± 6.82 years, and 2,996 (38.5%) suffering from depression. Moreover, there are 4,413 illiterates (56.0%), 1,416 urban residents (17.97%), 6,012 married individuals (76.29%), 749 living alone (9.51%), 548 with extremely poor self-rated health (6.95%), 5,917 with multimorbidity (75.09%), 4,450 not participating in any social activities (56.47%), 737 with a night sleep duration of over 8 h (9.35%), and 2,829 without a nap habit (35.9%). Differences between each variable are statistically significant (*P* < 0.001). Table 2 shows the specific basic characteristics.

**Table 2:** Basic characteristics of study participants (*n* = 7,880).

**Variables**	**Total (*n* = 7,880)**	**Non-depression (*n* = 4,884)**	**Depression (*n* = 2,996)**	***P***
Age, mean ± SD	69.05 ± 6.82	69.43 ± 7.23	68.44 ± 6.06	*P* < 0.001
Katz-ADL, mean ± SD	5.76 ± 0.91	5.74± 0.98	5.79 ± 0.76	*P* < 0.001
Lawton-IADL, mean ± SD	5.38 ±1.37	5.40 ± 1.45	5.35 ± 1.24	*P* < 0.001
Cognitive function, mean ± SD	19.31 ± 6.02	19.62 ± 6.20	18.83± 5.69	*P* < 0.001
**Gender**, ***n*** **(%)**				*P* < 0.001
Female	4,125 (52.35%)	2,259 (46.64%)	1,866 (61.46%)	
Male	3,755 (47.65%)	2,585 (53.36%)	1,170 (38.54%)	
**Self-rated health**, ***n*** **(%)**				*P* < 0.001
Very bad	548 (6.95%)	142 (2.93%)	406 (13.37%)	
Bad	1,578 (20.03%)	641 (13.23%)	937 (30.86%)	
Fair	4,275 (54.25%)	2,887 (59.6%)	1,388 (45.72%)	
Good	772 (9.8%)	596 (12.3%)	176 (5.8%)	
Very good	707 (8.97%)	578 (11.93%)	129 (4.25%)	
**Nighttime sleep**, ***n*** **(%)**				*P* < 0.001
< 6 h	3,273 (41.54%)	1,642 (33.9%)	1,631 (53.72%)	
6–8 h	3,870 (49.11%)	2,691 (55.55%)	1,179 (38.83%)	
>8 h	737 (9.35%)	511 (10.55%)	226 (7.44%)	
**Social events**, ***n*** **(%)**				*P* < 0.001
No	4,450 (56.47%)	2,732 (56.4%)	1,718 (56.59%)	
Yes	3,430 (43.53%)	2,112 (43.6%)	1,318 (43.41%)	
**Education level**, ***n*** **(%)**				*P* < 0.001
Illiterate	4,413 (56%)	2,509 (51.8%)	1,904 (62.71%)	
Elementary school	1,687 (21.41%)	1,079 (22.27%)	608 (20.03%)	
Junior high school and above	1,780 (22.59%)	1,256 (25.93%)	524 (17.26%)	
**Region**, ***n*** **(%)**				*P* < 0.001
City	1,416 (17.97%)	995 (20.54%)	421 (13.87%)	
Countryside	6,464 (82.03%)	3,849 (79.46%)	2,615 (86.13%)	
**Marital status**, ***n*** **(%)**				*P* < 0.001
Married	6,012 (76.29%)	3,769 (77.81%)	2,243 (73.88%)	
Divorce	95 (1.21%)	49 (1.01%)	46 (1.52%)	
Widowed	1,727 (21.92%)	993 (20.5%)	734 (24.18%)	
Unmarried	46 (0.58%)	33 (0.68%)	13 (0.43%)	
**Living alone**, ***n*** **(%)**				*P* < 0.001
No	7,131 (90.49%)	4,436 (91.58%)	2,695 (88.77%)	
Yes	749 (9.51%)	408 (8.42%)	341 (11.23%)	
**Naptime**, ***n*** **(%)**				*P* < 0.001
0 min	2,829 (35.9%)	1,638 (33.82%)	1,191 (39.23%)	
< 30 min	1,158 (14.7%)	702 (14.49%)	456 (15.02%)	
30–90 min	2,208 (28.02%)	1,387 (28.63%)	821 (27.04%)	
>90 min	1,685 (21.38%)	1,117 (23.06%)	568 (18.71%)	
**Smoke**, ***n*** **(%)**				*P* < 0.001
No	4,644 (58.93%)	2,692 (55.57%)	1,952 (64.3%)	
Yes	3,236 (41.07%)	2,152 (44.43%)	1,084 (35.7%)	
**Frequency of physical activity**, ***n*** **(%)**				*P* < 0.001
0 times a week	1,138 (14.44%)	726 (14.99%)	412 (13.57%)	
1–4 times a week	1,121 (14.23%)	670 (13.83%)	451 (14.86%)	
More than 4 times a week	5,621 (71.33%)	3,448 (71.18%)	2,173 (71.57%)	
**Multimorbidity**, ***n*** **(%)**				*P* < 0.001
No	1,963 (24.91%)	1,437 (29.67%)	526 (17.33%)	
Yes	5,917 (75.09%)	3,407 (70.33%)	2,510 (82.67%)	
**Drinking**, ***n*** **(%)**				*P* < 0.001
No	5,365 (68.08%)	3,147 (64.97%)	2,218 (73.06%)	
Yes	2,515 (31.92%)	1,697 (35.03%)	818 (26.94%)	

### Predicting depression in Chinese older adults using the ML models

The SMOTE algorithm was used to oversample the minority samples. Thereafter, the dataset was partitioned into training and test sets at a 6:4 ratio. The model was trained on the training set, and its ability to generalize was evaluated on the test set. Using the RF algorithm as the classification model, recursive feature elimination was performed on the training set. Three-fold cross-validation was used to derive AUC area scores from various feature selection combinations, in which 16 was identified as the optimal number of features. According to the rank-sum test, no significant difference was noted in the continuous variables between the training and test sets (*P* > 0.05). The chi-square test also indicated no significant difference for the other categorical variables (*P* > 0.05).

**Figure 2:**
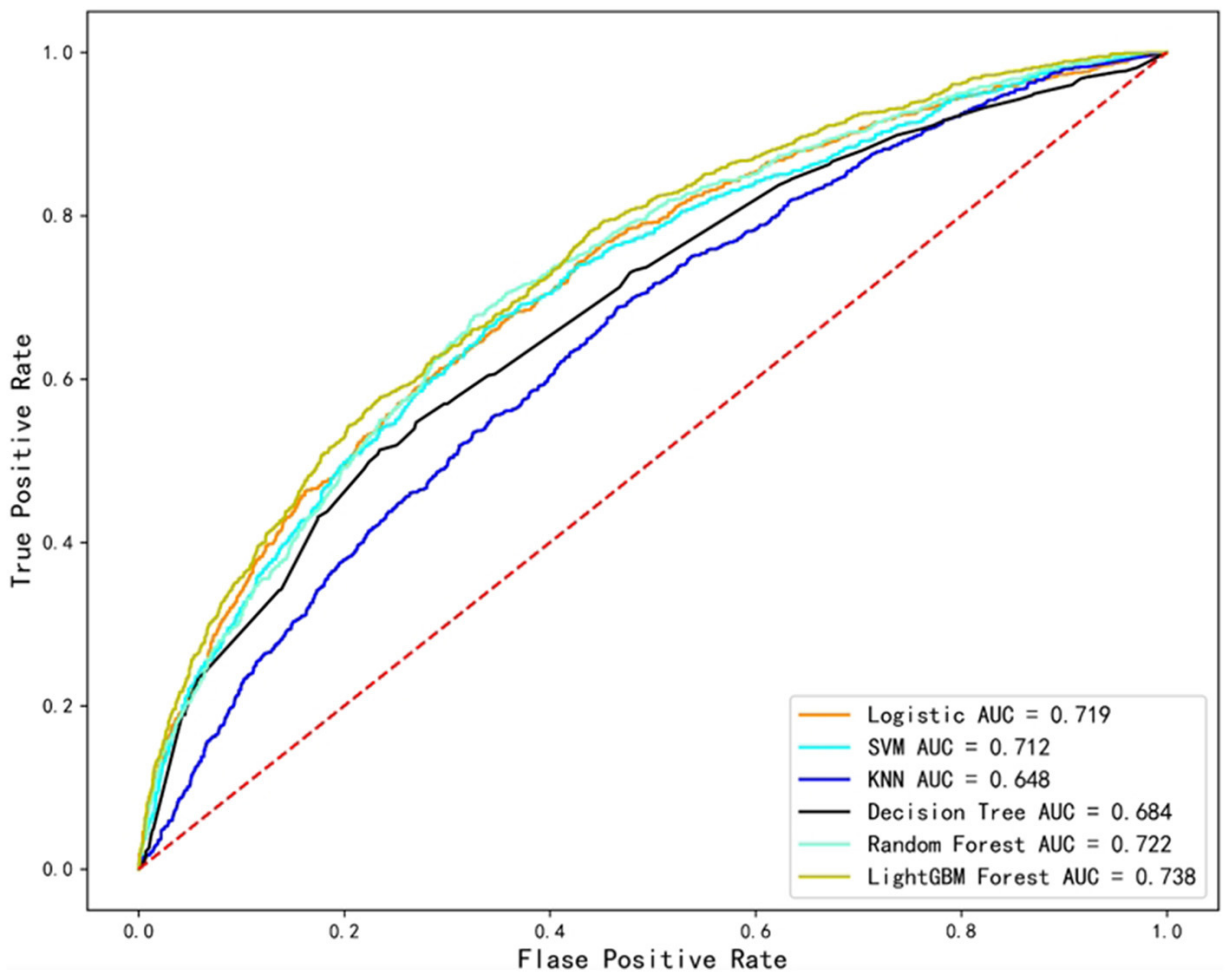
Predictive performance of six machine learning models for depression in older adults. ROC curve (the *x*-axis indicates the false positive rate, and the *y*-axis represents the true positive rate).

For depression prediction, KNN and LightGBM had the lowest (AUC = 0.647) and highest (AUC = 0.738) AUC values, respectively, among the six ML models (Figure 2). Moreover, the Delong statistical comparison indicated that the performance of the different models' ROC curves vary (*P* < 0.01, Table 3).

**Table 3:** Prediction performance of depression in Chinese older adults using six machine learning models.

**Model**	**AUC**	***P*-value**	**Accuracy**	**Specificity**	**PPV**	**NPV**	**Brier score**
Logistic regression	0.719	Reference	0.662	0.641	0.749	0.543	0.211
KNN	0.647	< 0.001	0.623	0.497	0.698	0.498	0.239
Decision tree	0.684	< 0.001	0.663	0.519	0.722	0.554	0.223
SVM	0.712	< 0.001	0.676	0.577	0.743	0.567	0.209
Random forest	0.722	< 0.001	0.679	0.570	0.742	0.573	0.205
LightGBM	0.738	< 0.001	0.696	0.553	0.744	0.604	0.198

AUC, area down the receiver operating curve; PPV, positive predictive value; NPV, negative predictive curve. *P*-value is the results of Delong test of AUC curve of different machine learning models compared with logistic regression model.

**Figure 3:**
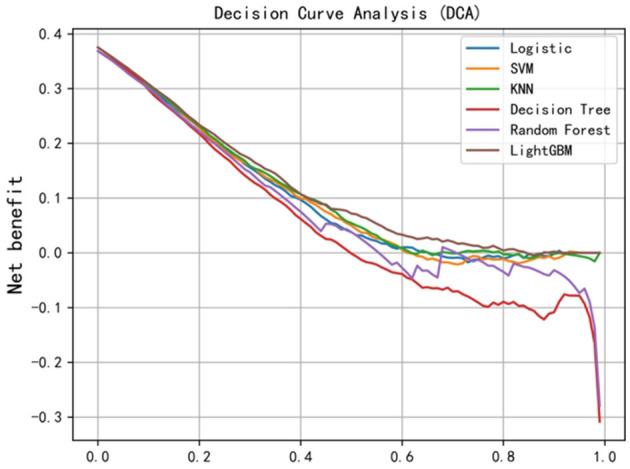
DCA curve (the *x*-axis indicates the threshold probability, and the *y*-axis represents net benefit).

According to the DCA curve (Figure 3), if the threshold probability of depression among Chinese older adults exceeds 0, then the LightGBM model outperforms the other models in the 0.00–0.85 range. From the results, the LightGBM model's Brier score was 0.198, which was the lowest among all models. Therefore, the best performance was demonstrated by the LightGBM model.

### Feature importance and variables interpretation

**Figure 4:**
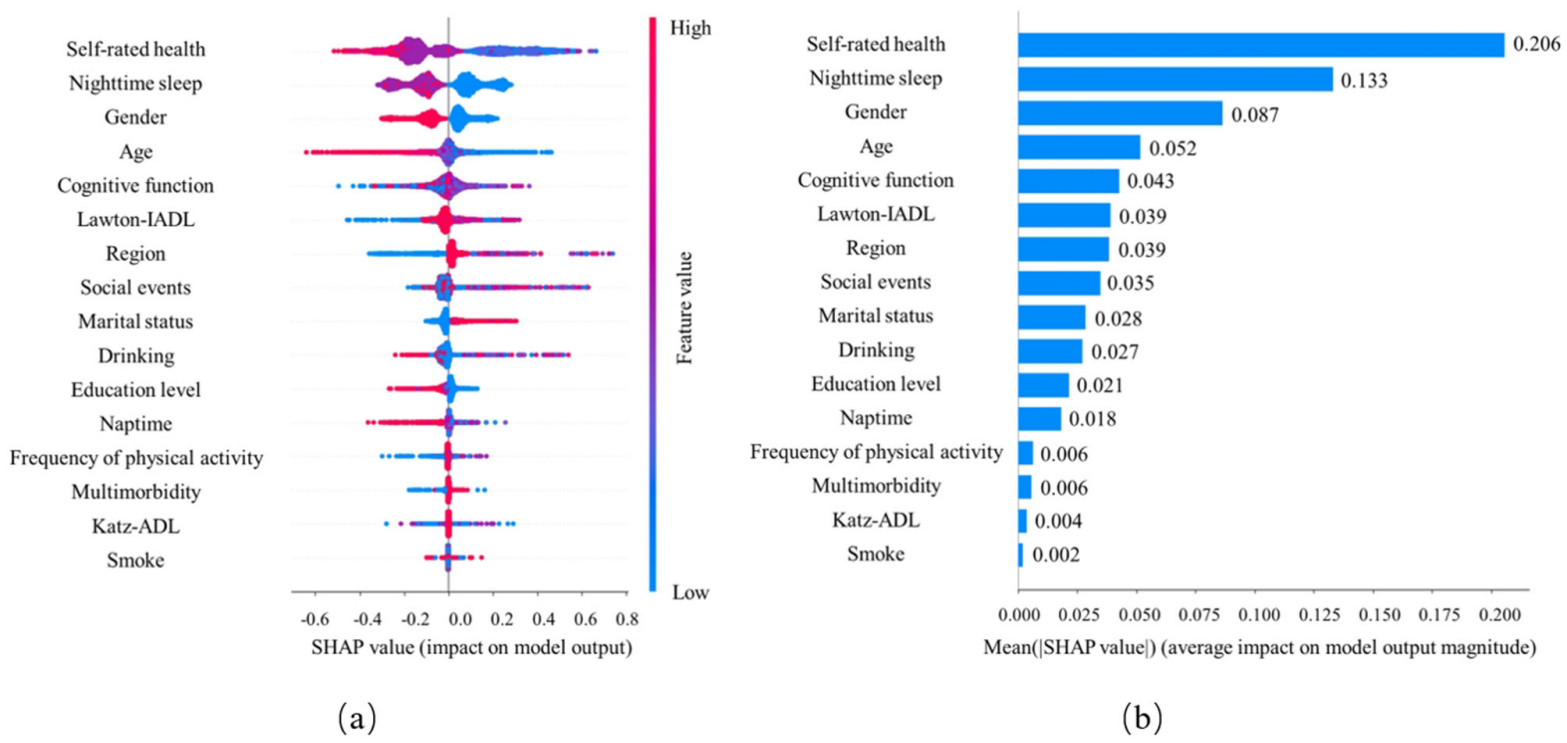
Visual explanation of predictive behavior of depression in older adults based on machine learning prediction models. **(A)** Each dot represents an individual prediction, dot's position on the x-axis shows the impact that predictor has on the model's prediction for that individual. When multiple dots land on the same x position, they pile up to show density. The color of the dot represents the level of the predictor related to that individual (color reference on the tight). **(B)** Bar chart of average feature importance based on SHAP value magnitude.

In depression prediction, the LightGBM model ranks feature importance (see Figure 4). The model indicates that the five most important features for predicting depression are self-assessment of health, nighttime sleep, gender, age, and cognitive function. Note that self-rated health, nighttime sleep, and age are negatively correlated with depression occurrence. Moreover, older women have a higher probability of experiencing depression than older men. The findings are significant for early identification and intervention in depression.

## Discussion

### Major findings

The newly released 2020 CHARLS data was used by the current research to conduct a depression-prediction task on the older adult under the decision support of six ML algorithms. For feature selection recursive feature elimination, the RF algorithm was used as classification model. Meanwhile, constructing and validating the model feature the use of 16 important feature variables. According to the results, the LightGBM model shows the best performance in terms of the AUC value and outperforms other models in predictive performance. The important contributions of the model prediction results are explained by the SHAP analysis, indicating that the five most important features for predicting depression are self-rated health, nighttime sleep, gender, age, and cognitive function. The contribution of each feature to the model prediction results is intuitively presented by the summary graph. The findings present the key factors affecting the occurrence of depression, which is critical in the early identification and intervention of depression.

### Identifying key predictors of depression in the older adult

Social demographic factors, health status, behavioral factors, mental health factors, and other dimensions of the older adult are the variables selected in this study. Previous research has studied these indicators for their impact on depression, thereby showing the credibility and validity of the selected predictive factors. As a predictive factor, self-rated health contributes the most, which is consistent with a previous study that has considered this factor as the most important predictive factor for depression in the older adult (39). Moreover, numerous previous studies have indicated that a lower level of self-rated health is a risk factor for the occurrence of depression among the older adult (40–42). Previous research has likewise shown relationship between depression and the older adult's sleep duration (43). Sleep duration and depression have a *U*-shaped relationship, and the likelihood of depression is lowest for the older adult sleeping 6– 8 h at night (44). The result of a meta-analysis indicates that considered risk factors of depression are short and long sleep durations (45). Happiness can increase with an increase in sleep duration, which is closely related to a reduction in the occurrence of depression (46). A sharp decrease in sleep duration is associated with an increase in unhealthy behaviors such as obesity, smoking, and drinking, which can affect the older adult's psychology (47). Excessive sleep duration (>9 h) is associated with depression (48). A decrease in physical activity is associated with excessive sleep duration, which may reduce the production of key hormones that regulate emotions (e.g., serotonin or dopamine), thereby increasing the likelihood of depression among the older adult (49).

According to the current research, older adult women are likely to suffer from depression compared with older adult men. This finding is consistent with most previous studies' outcomes (50–53). For the longest time, women have been found to have a higher prevalence of depression. Epidemiological studies have indicated that the pervasiveness of depression among women is nearly twice that of men. Genetically determined susceptibility, hormone fluctuations related to various aspects of reproductive function, and hypersensitivity to hormone fluctuations mediating depressive states are among the reasons for this phenomenon (54). However, other findings have shown lack of association between gender and the occurrence of depression (55–57). The most common predictive factors for the occurrence of depression among the older adult are age, physical health, and cognitive function (18). According to previous studies, age is a risk factor for the occurrence of depression among the older adult (58–60), or it is not associated with the occurrence of depression (61–63). The current research presents a novel finding that age is negatively correlated with the occurrence of depression, which deviates from a few previous research results. This outcome possibly shows that during the COVID-19 pandemic, age might be associated with other protective factors (e.g., life experience and coping strategies), thereby potentially assisting in the reduction of depression risks (64). Accordingly, age may be a protective factor (65).

The relationship between cognitive function and depression remains mired in controversy. Two studies have shown the lack of effect of cognitive impairment on the depressed population (66, 67). Meanwhile, two other studies have reported that the risk of depression among the older adult increases owing to cognitive impairment (68, 69). Researchers have analyzed data from 8,382 men and women aged at least 65 years who participated in the US Health and Retirement Study from 1998 to 2010. Low depression and high depression categories are associated with rapid cognitive decline and estimated impact after adjusting for the interaction of depression and time (70).

### Optimizing older adult depression prediction with lightGBM and SHAP

According to 20 studies that systematically evaluated the prediction model of older adult depression risk, 55% of the prediction models were built using ML technology, while seven models (35%) used multivariate logistic regression and two models (10%) used linear regression (18). First, to establish a prediction model for the occurrence of depression in the older adult, the current study used the SMOTE sampling technique to balance the “depression” sample ratio of 0 and 1. Accordingly, the model's prediction accuracy and stability are improved by balancing the number of older adult people in the dataset's different categories. Second, AUC is the indicator most commonly used for evaluating the prediction model's performance, while DCA is likewise utilized to assess the model's clinical value and has been applied in numerous medical studies, showing immense clinical utility (71). This study's results indicate that the LightGBM model performs best in terms of the AUC value. The possible reason is that LightGBM can handle numerous features and has good handling ability for unbalanced datasets. This model is further assessed using the DCA method. The net benefit of LightGBM is the largest when the net return is compared on the decision curve. If depression's threshold probability surpasses 0, then the performance of the LightGBM model is better than that of the other models in the 0.00–0.85 range. This finding is crucial because it indicates that the LightGBM model has excellent performance under most clinical decision thresholds. The LightGBM model's The Brier score is 0.198, the lowest among all models. The Brier score is an indicator to measure prediction accuracy. The lower the value, the higher the accuracy of prediction, thereby further confirming the LightGBM model's superiority.

By contrast, complex patterns and interactions are revealed by ML methods; however, these methods are often criticized for their “black box” nature. Accordingly, the SHAP technology can be used to solve this problem. The reason is that they can empirically indicate the importance of each feature to the model output, thereby enabling the combination of features and non-linear interactions, while providing insights into the current relationships among variables, which are considered correlations instead of causal relationships. Prior studies have successfully utilized SHAP values for ML interpretation in medical research (72). Ballester et al. analyzed the suicide risk of young people by using the gradient tree boosting model and SHAP values (73). Although the aforementioned method has been utilized in various fields of ML interpretation, to our knowledge, the SHAP value has yet to be applied to investigate the predictive research of depression's occurrence among the older adult in China. Therefore, this study's results can present a reference for enhancing the prediction model of depression's occurrence among the older adult in China.

## Limitations

Several limitations characterize this research. First, the current study only uses CES-D to evaluate depression's symptoms among the older adult. While CES-D is not the gold standard technique for depression diagnosis, which may lead to bias in the evaluation results, the CESD-10 tool has been validated in the Chinese population and shows good validity, potentially improving the credibility and validity of the results. Second, this research is based on cross-sectional data, preventing the determination of the causal direction of the observed associations. Longitudinal studies are needed to establish causal relationships. Third, the data survey was conducted during the COVID-19 pandemic in 2020. This unique stressor may have influenced both predictors (e.g., sleep patterns) and the outcome (depression), acting as a collider and introducing bias into the model. New data after the pandemic have not yet been released, and a new longitudinal data model can be constructed subsequent to the data release post-pandemic. Fourth, reporting bias is possible from the study participants because self-reported data may not always accurately reflect their true state of health and wellbeing. Participants may underreport or overreport their symptoms due to social desirability or recall bias. Information bias could also occur due to inaccuracies in the collected data, which may affect the results. Specifically, the 2020 CHARLS data may have inaccuracies due to data entry errors, participants' misinterpretation of survey questions, or inconsistencies in data collection processes across different regions. Fifth, variables such as gender, age, and cognitive function, while strong predictors of depression, may correlate with other unmeasured factors like socioeconomic status or life stress, potentially confounding the results. Cognitive impairment might indirectly reflect life-stage factors or cumulative health burdens and also be a symptom of depression, particularly in older populations (“pseudo-dementia”). Self-rated health, identified as the most significant predictor, could act as a proxy for unmeasured variables such as chronic disease burden or healthcare access, leading to residual confounding. Sixth, the use of the SMOTE algorithm to oversample minority depression cases helps balance the dataset but may inadvertently create artificial correlations or inflate the importance of less influential variables, introducing collider bias. Lastly, although measures have been taken to ensure the good performance of the model, it still necessitates validation with an independent external cohort once it is established. This step is crucial to confirm the model's generalizability and robustness. Notwithstanding these limitations, the current research indicates that the constructed model can be utilized for predicting and providing further interventions for the occurrence of depression among the older adult.

## Conclusion

This research uses six ML algorithms to build a prediction model for the occurrence of depression among the older adult in China. Moreover, the LightGBM algorithm-based predictive model has superior comprehensive performance compared with other models. Therefore, the research results can provide a theoretical reference for the accurate identification of high-risk groups from multiple dimensions and the implementation of the necessary early interventions. The proposed model identifies key influencing factors (i.e., self-rated health, nighttime sleep duration, gender, age, and cognitive function) and some potential influencing factors. These factors should be continuously enhanced in future model construction research.

## Data Availability

The datasets presented in this study can be found in online repositories. The names of the repository/repositories and accession number(s) can be found below: (China Health and Retirement Longitudinal Study, CHARLS) 2020 Data Access Links: https://charls.charlsdata.com/pages/data/111/zh-cn.html. If the database is not available, you can obtain the data directly from the corresponding author.
